# The Na,K-ATPase-Dependent Src Kinase Signaling Changes with Mesenteric Artery Diameter

**DOI:** 10.3390/ijms19092489

**Published:** 2018-08-23

**Authors:** Lin Zhang, Christian Aalkjaer, Vladimir V. Matchkov

**Affiliations:** 1 Department of Exercise Physiology, Beijing Sport University, Beijing 100084, China; zhanglinbsu@126.com; 2 Department of Biomedicine, Health, Aarhus University, DK-8000 Aarhus C, Denmark; ca@biomed.au.dk

**Keywords:** Na,K-ATPase, Src, intracellular calcium sensitization, arterial contraction

## Abstract

Inhibition of the Na,K-ATPase by ouabain potentiates vascular tone and agonist-induced contraction. These effects of ouabain varies between different reports. In this study, we assessed whether the pro-contractile effect of ouabain changes with arterial diameter and the molecular mechanism behind it. Rat mesenteric small arteries of different diameters (150–350 µm) were studied for noradrenaline-induced changes of isometric force and intracellular Ca^2+^ in smooth muscle cells. These functional changes were correlated to total Src kinase and Src phosphorylation assessed immunohistochemically. High-affinity ouabain-binding sites were semi-quantified with fluorescent ouabain. We found that potentiation of noradrenaline-sensitivity by ouabain correlates positively with an increase in arterial diameter. This was not due to differences in intracellular Ca^2+^ responses but due to sensitization of smooth muscle cell contractile machinery to Ca^2+^. This was associated with ouabain-induced Src activation, which increases with increasing arterial diameter. Total Src expression was similar in arteries of different diameters but the density of high-affinity ouabain binding sites increased with increasing arterial diameters. We suggested that ouabain binding induces more Src kinase activity in mesenteric small arteries with larger diameter leading to enhanced sensitization of the contractile machinery to Ca^2+^.

## 1. Introduction

The Na,K-ATPase is strongly suggested to be implicated in hypertension [1]. A prolonged inhibition of the Na,K-ATPase by cardiac glycosides, for example, ouabain, leads to chronic elevation of blood pressure in rodents [2,3,4,5,6]. This can be prevented by the ouabain antagonist rostafuroxin or by an ouabain-binding antibody [7,8]. It is important from a clinical perspective since endogenous ouabain is synthesized in the zona glomerulosa cells of the adrenal cortex and levels of ouabain correlate positively with blood pressure in patients [9]. The mechanism behind ouabain-induced elevation of blood pressure is not fully understood [9,10] but the vascular wall serves as a main target for ouabain. Hemodynamic changes induced by ouabain are in line with the conventional view on hypertension pathology, where elevated blood pressure is mediated by an increase in peripheral vascular resistance [11,12].

Two isoforms of the Na,K-ATPase are present in the vascular wall, namely, the α1- and α2-isoforms [13]. In rodent, the isoenzyme containing the α1-isoform has an approximately 100-times lower affinity for ouabain than the Na,K-ATPase α2-isoform [14]. These two isoforms have also been ascribed different localizations and functions [15], where the Na,K-ATPase α2 isoform is involved in regulation of vascular tone [5,16,17,18,19,20,21] and, thus, regulation of blood pressure [1]. Thus, ouabain in concentrations up to 10 µM inhibits preferentially the Na,K-ATPase α2 isoform and potentiates vascular tone [18,20,22] in vivo and in vitro. These concentrations of ouabain have also been reported to potentiate agonist-induced arterial contraction [22,23,24,25,26,27]. It has been suggested to be a result of ouabain-induced depolarization leading to an increase in voltage-dependent Ca^2+^ influx, although an effect from the Na^+^,Ca^2+^-exchanger inhibition via functional interaction of these two membrane transporters is likely also of importance [23,28,29].

This cannot however be the only mechanism responsible for pro-contractile action of ouabain [15]. Thus, a α2-isoform-dependent modulation of intracellular calcium ([Ca^2+^]_i_) homeostasis is not consistent with unchanged steady-state [Ca^2+^]_i_ and [Na^+^]_i_ in spite of potentiated agonist-induced contraction [16,22,23,24,25,30]. Moreover, inhibition of the Na,K-ATPase by digoxin does not raise blood pressure and antagonizes the pro-hypertensive action in ouabain [10,29] suggesting an importance of ion-transport-independent ouabain signaling. Consistent with this, we [22,25,31] and others [29] have shown that ouabain modulates intracellular signaling and agonist-induced contraction of smooth muscles, as least in part, via Src kinase activation. We have shown that, similar to other cell types [32,33,34,35,36,37,38], low concentrations of ouabain phosphorylate smooth muscle Src kinase at Y418 [31]. This leads to the sensitization of the contractile apparatus to Ca^2+^ via the myosin phosphatase targeting protein 1 (MYPT1) phosphorylation [22,25].

The activity of Src is relatively high in vascular smooth muscle cells and has previously been suggested to be important for mesenteric artery agonist-induced contraction [39]. However, the significance of the Na,K-ATPase-dependent Src signaling in arteries of different diameter might vary significantly. This can be at least part of the reason for the variable effect of ouabain on agonist-induced arterial contraction [22,24,40]. This variability can also be important for modulation of peripheral resistance in vivo, where terminal arterioles are important under resting conditions, while principle arteries increase their contribution to hemodynamic resistance during sympathetic excitation [41].

In this study, we assessed whether the potentiating effect of ouabain on noradrenaline-induced contraction is different in mesenteric small arteries of different diameters and whether this is due to variable Src-dependent Ca^2+^ sensitization of the contractile apparatus. We further assessed whether this difference is due to variation in the expression of either Src kinase or the high-affinity Na,K-ATPase in mesenteric arteries of different diameters.

## 2. Results

**Figure 1:**
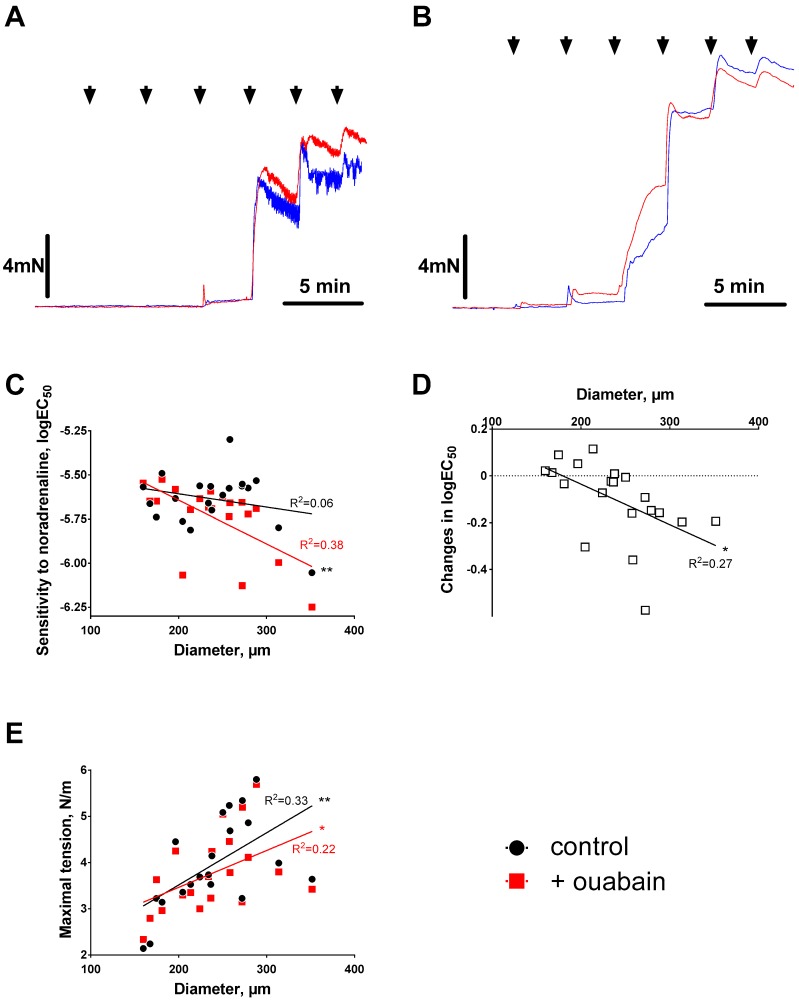
Effect of ouabain on agonist-induced contraction varies with arterial diameter. (**A**,**B**) show representative concentration–response curves for noradrenaline-induced contractions of mesenteric arteries before (blue) and after 15 min incubation with 10 µM ouabain (red). Inner diameter of the artery in A was 167 µm, and the artery in B–314 µm. Noradrenaline was applied in concentrations of 0.1, 0.3, 1, 3, 10, and 30 µM as indicated by arrows. Sensitivity of arteries to noradrenaline was expressed as logEC_50_ (in (**C**) and was measured for each arterial segment under control conditions and in the presence of ouabain. Ouabain-induced changes in the sensitivities are shown in (**D**) as the difference between sensitivities in the presence of ouabain and under control conditions. (**E**) shows maximal tension responses to 30 µM noradrenaline under control conditions and in the presence of ouabain. Values for goodness-of-fit of linear regression (R^2^) are indicated by the lines. *p* < 0.05 (*) and *p* < 0.01 (**), *n* = 20.

Mesenteric small arteries of different diameter were constricted with increasing concentrations of noradrenaline (Figure 1A,B). Under control conditions, there was no significant correlation between sensitivities to noradrenaline (i.e., logEC_50_) and arterial diameters (Figure 1C). After pre-incubation with 10 µM ouabain, the sensitivity to noradrenaline significantly increased with increasing arterial diameter (Figure 1C,D). Maximal contraction increased with increase in arterial size too but in contrast to noradrenaline sensitivity, this was seen both under control conditions and in the presence of ouabain (Figure 1E).

**Figure 2:**
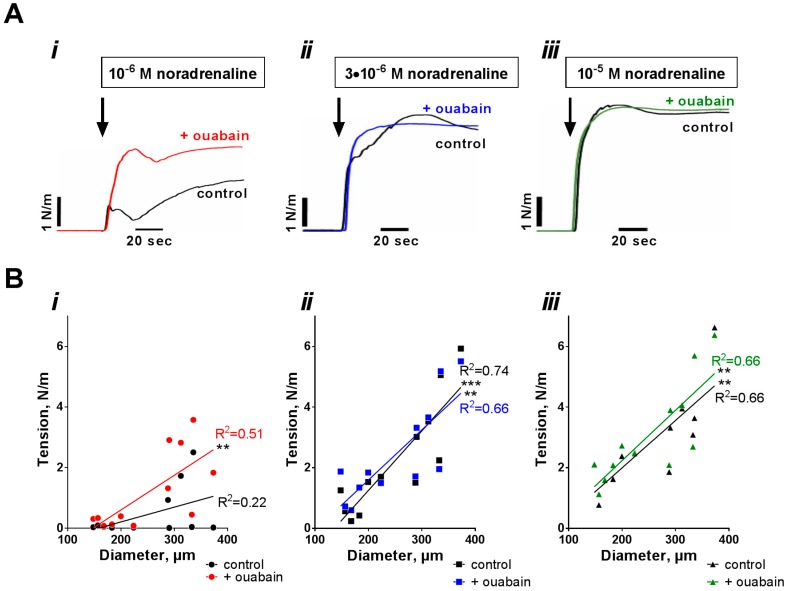
The potentiating effect of ouabain on noradrenaline-induced contraction depends on arterial diameter. Wall tension changes of mesenteric arteries of different diameters was assessed in response to stimulation with either 1 µM (**i**), 3 µM (**ii**) or 10 µM (**iii**) noradrenaline. Note, these wall tensions were measured simultaneously with [Ca^2+^]_i_, as shown in Figure 3. The stimulations were then repeated after 15 min incubation with 10 µM ouabain. Representative curves for wall tension responses are shown in (**A**). (**B**) shows averaged noradrenaline-induced changes in wall tension correlated with arterial diameter. Linear regression quantifies goodness of fit with R^2^ as indicated by the lines. *p* < 0.01 (**) and *p* <0.001 (***), *n* = 12.

**Figure 3:**
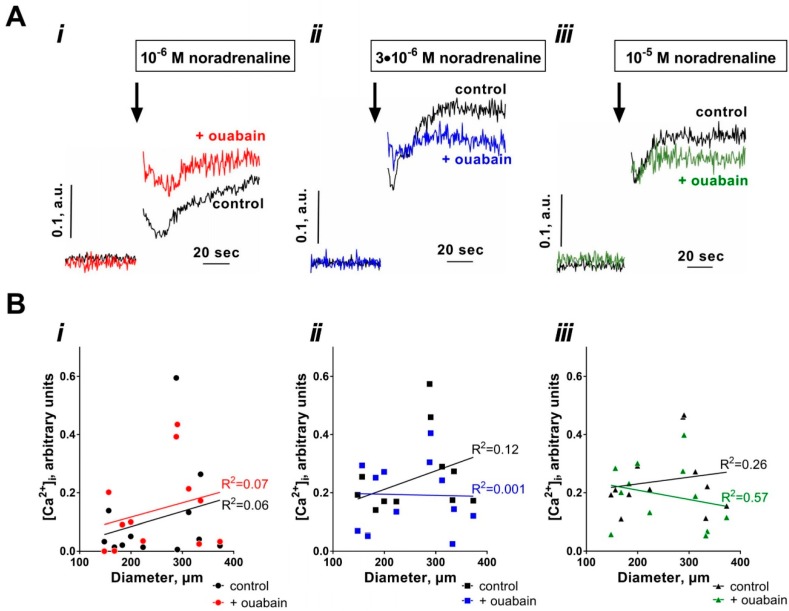
Noradrenaline-induced changes in intracellular calcium ([Ca^2+^]_i_) do not correlate with arterial diameter. Mesenteric arteries of different diameters were loaded with the Ca^2+^-sensitive dye (Fura-2/AM) to access wall tension (showed in Figure 2) and [Ca^2+^]_i_ changes in response to stimulation with either 1 µM (**i**), 3 µM (**ii**) or 10 µM (**iii**) noradrenaline. Representative curves for [Ca^2+^]_i_ are shown in (**A**). (**B**) shows that averaged noradrenaline-induced changes in [Ca^2+^]_i_ did not correlate with arterial diameter. Linear regression quantifies goodness of fit with R^2^ as indicated by the lines. No significant correlation was found. *n* = 12.

To analyze whether the observed diameter-dependent effect of ouabain on the sensitivity to noradrenaline was due to difference in the sensitization of contractile apparatus to [Ca^2+^]_i_ or due to difference in [Ca^2+^]_i_, we performed simultaneous measurements of wall tension and [Ca^2+^]_i_ changes in response to noradrenaline stimulation (Figure 2 and Figure 3). Arteries were stimulated by a single concentration of noradrenaline (either 1 µM, or 3 µM, or 10 µM) under control conditions and after pre-incubation with ouabain (Figure 2A and Figure 3A). Contraction to 1 µM noradrenaline did not correlate with arterial diameter under control conditions but there was a positive correlation in the presence of ouabain (Figure 2(Bi)). In contrast, an increase in tension in response to 3 µM and 10 µM noradrenaline increased with arterial caliber similar both under control conditions and in the presence of ouabain, that is, there was no potentiating effect of ouabain (Figure 2(Bii,iii)). Intracellular calcium ([Ca^2+^]_i_) raised in response to noradrenaline similarly in arteries of different diameter and, thus, no significant correlation between changes in [Ca^2+^]_i_ and arterial diameter was seen at any concentration of noradrenaline (Figure 3).

**Figure 4:**
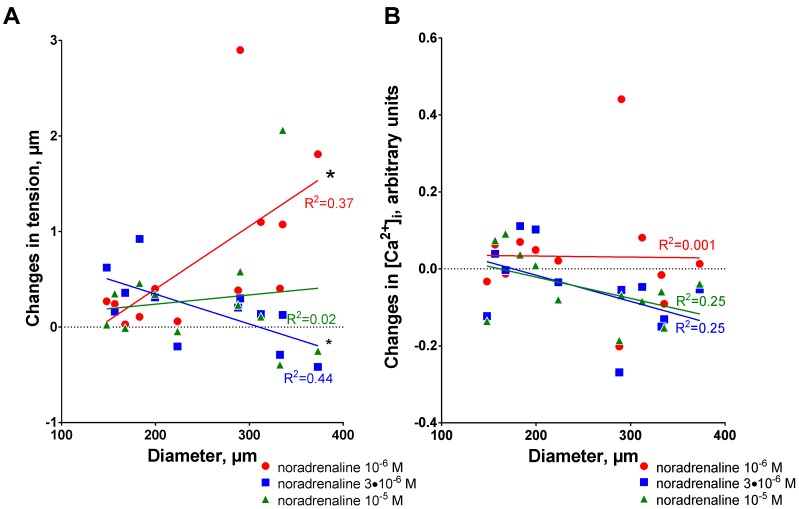
The potentiating effect of ouabain increases with arterial diameter for wall tension responses to 1 µM noradrenaline (**A**) but not for the changes in [Ca^2+^]_i_ (**B**). (**A**) shows the differences in wall tension increase to 1 µM (red), 3 µM (blue) and 10 µM (green) noradrenaline stimulations after incubation with 10 µM ouabain, the data are shown in Figure 2B. (**B**) demonstrates the corresponding differences in [Ca^2+^]_i_ changes after incubation with 10 µM ouabain, the data are shown in Figure 3B. Linear regression quantifies goodness of fit with R^2^, as indicated by the lines. * indicates significance of the correlation, where *p* < 0.05, *n* = 12.

Accordingly, we found a significant correlation between arterial diameter and ouabain-dependent increase in tension in response to 1 µM noradrenaline (Figure 4A). In contrast, a negative correlation was seen for 3 µM noradrenaline stimulation and no correlation was seen for 10 µM noradrenaline (Figure 4A). No correlation between arterial diameter and ouabain-induced changes in [Ca^2+^]_i_ responses was seen at all noradrenaline concentrations (Figure 4B).

**Figure 5:**
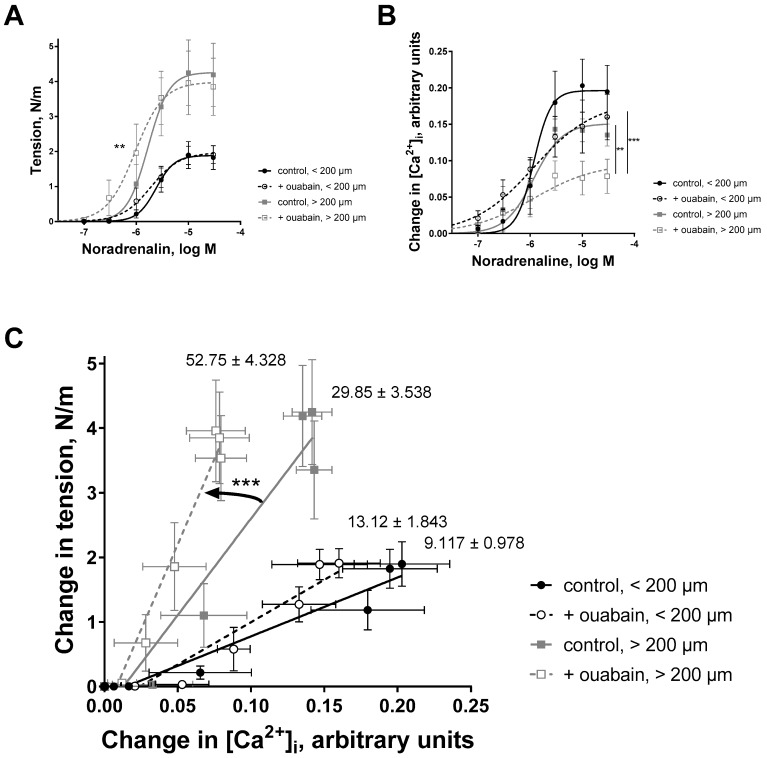
Ouabain has a stronger sensitizing effect on the arteries of large diameter compared to small arteries. Mesenteric arteries were subdivided into two groups; one group consisted arteries with inner diameter below 200 µm and another group consisted arteries of inner diameter above 200 µm. Arteries were loaded with the Ca^2+^-sensitive dye (Fura-2/AM) and simultaneous measurements in wall tension (**A**) and [Ca^2+^]_i_ (**B**) were obtained in the presence of different noradrenaline concentrations. (**C**) shows the changes in wall tension as a function of the changes in [Ca^2+^]_i_ for different groups under control conditions and in the presence of 10 µM ouabain, i.e., the data re-plotted from (**A**,**B**). The slopes of tension–[Ca^2+^]_i_ relations are indicated as values on the plot. Note, that the steepness of the tension–[Ca^2+^]_i_ relation was significantly increased for arteries with inner diameter above 200 µm. *p* < 0.01 (**) and *p* < 0.001 (***), *n* = 4–5.

We sub-divided arteries to groups with smaller (inner diameter < 200 µm) and larger (>200 µm) diameters to analyze their noradrenaline concentration-dependent responses in wall tension (Figure 5A) and in [Ca^2+^]_i_ (Figure 5B). When tension was plotted as a function of [Ca^2+^]_i_, the slope for arteries with larger diameter was significantly steeper (Figure 5C). This steepness (i.e., sensitivity to Ca^2+^) was further significantly increased in the presence of ouabain suggesting additional sensitization to Ca^2+^. This was not the case for the group of smaller arteries (Figure 5C). 

**Figure 6:**
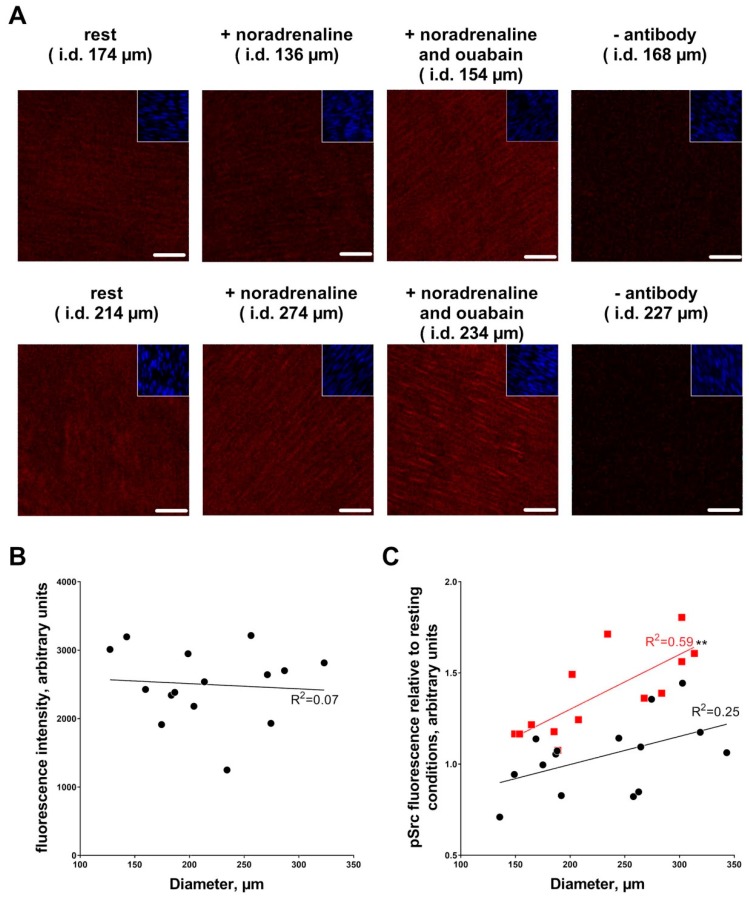
Ouabain-induced phosphorylation of Src kinase increases with arterial diameter. Arteries were fixed at resting conditions, after stimulation with 1 µM noradrenaline or incubation with 10 µM ouabain with the following 1 µM noradrenaline stimulation, as indicated in representative images ((**A**); bars indicates 20 µm). A phosphorylated Src (pSrc) related fluorescence intensity was measured in smooth muscle layer in the vascular wall, as indicated by SYTO-16 based nuclei staining (inserts in images in (**A**)). No difference in fluorescence intensity in arteries with different inner diameter was seen under resting conditions (**B**). In 15 experiments, the fluorescence intensity of the artery under resting conditions was compared to the intensity in the artery after noradrenaline stimulation with and without pre-incubation with 10 µM ouabain. There was no correlation between phosphorylated Src and inner diameter for noradrenaline stimulated arteries but in the presence of ouabain phosphorylated Src was significantly *p* < 0.01 (**) increased with arterial diameter. Linear regression quantifies goodness of fit with R^2^, as indicated by the graphs.

Arteries of different diameter had similar level of Y418-phosphorylated Src kinase in the wall media (Figure 6B). No correlation of Src kinase phosphorylation with arterial diameter was seen in the presence of 1 µM noradrenaline (Figure 6C). However, in the presence of ouabain, Src kinase phosphorylation positively correlated with arterial diameter (Figure 6C).

**Figure 7:**
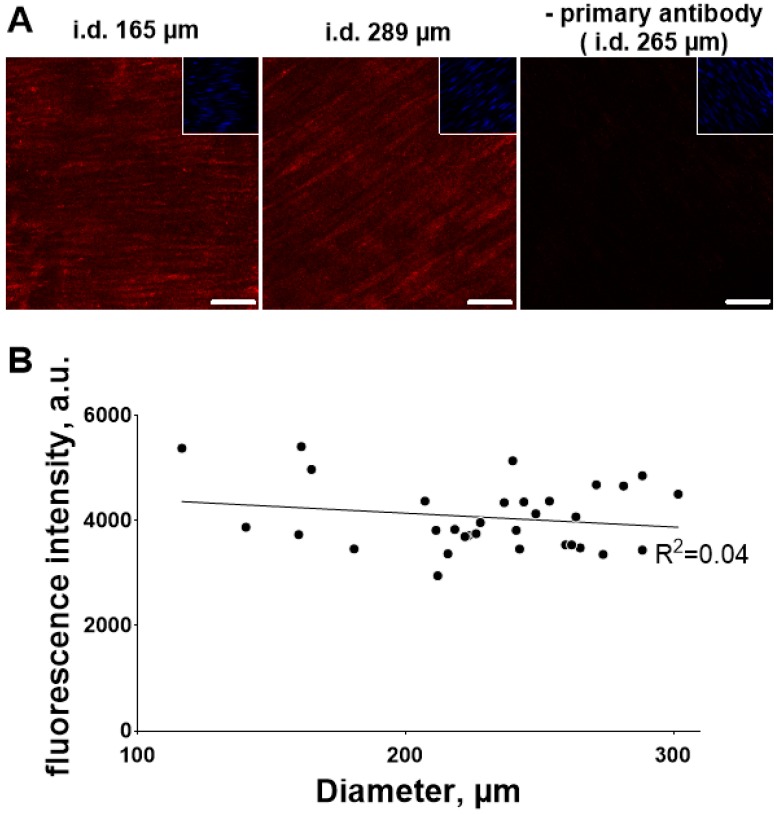
No difference in total Src expression between mesenteric arteries with different diameters. (**A**) shows representative images of arteries with different diameters as indicated (bars indicates 20 µm). Total Src related fluorescence intensity was measured in smooth muscle layer in the vascular wall, as indicated by SYTO-16 based nuclei staining (inserts in images). No difference in fluorescence intensity in arteries with different inner diameter was seen (**B**). R^2^ on the graph indicates linear regression quantifies goodness of fit. *n* = 32.

We have tested whether this difference in Src phosphorylation is due to difference in total Src kinase expression in the arteries with different diameter. No significant difference in total Src expression in the arteries with different diameter was found (Figure 7).

**Figure 8:**
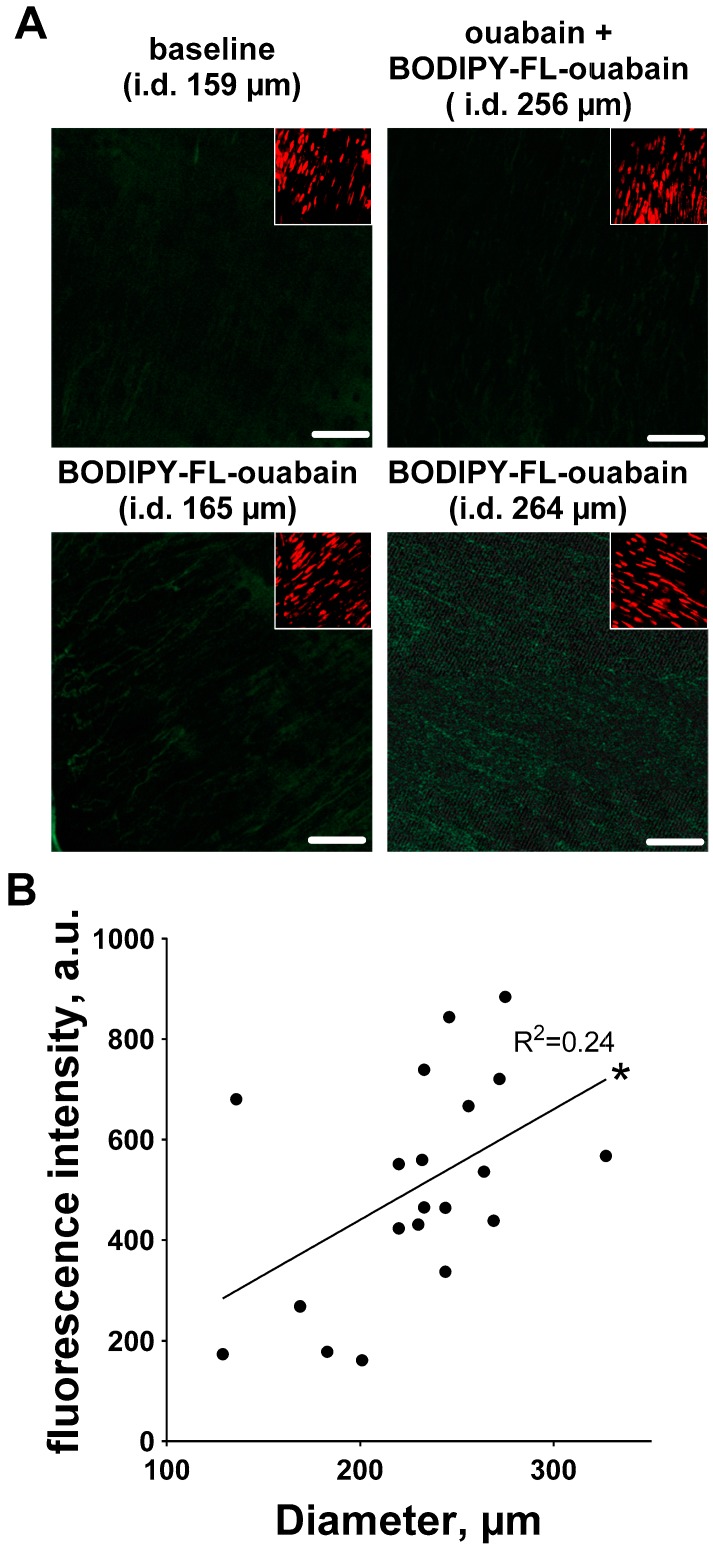
Increase in arterial diameter is associated with an increased density of the high-affinity ouabain binding sites. Arterial segments of different diameter were incubated with 10 µM BODIPY FL ouabain. (**A**) shows representative images of arterial segment without pharmacological intervention (i.e., baseline), two arterial segments of different diameters as indicated treated with 10 µM BODIPY FL ouabain, and artery first pre-treated with 1 mM ouabain and then exposed to 10 µM BODIPY FL ouabain (bars indicates 20 µm). Fluorescence intensity was measured in smooth muscle layer in the vascular wall, as indicated by SYTO-82 based nuclei staining (inserts in images). BODIPY FL ouabain related fluorescence intensity increases with an increase in arterial inner diameter (**B**). R^2^ on the graph indicates linear regression quantifies goodness of fit. *n* = 20, *p* < 0.05 (*).

We have also tested whether the density of high-affinity ouabain binding sites is different in arteries with different diameters. Experiments with BODIPY FL ouabain indicate that the number of high-affinity ouabain binding sites increases with arterial diameter (Figure 8).

## 3. Discussion

One of the main findings of this study is that ouabain-induced potentiation of the sensitivity to noradrenaline correlates positively with an increase in diameter of rat mesenteric small arteries from ~150 µm to ~350 µm. To understand the molecular mechanism responsible for this, we assessed [Ca^2+^]_i_ and Src kinase phosphorylation in these arteries when exposed to ouabain. From these experiments we conclude that the diameter-dependent effect of ouabain was not due to differences in [Ca^2+^]_i_ responses in the vascular smooth muscle cells but due to sensitization of their contractile machinery to [Ca^2+^]_i_. This was associated with Src kinase phosphorylation, that is, Src activation, which correlated positively with arterial diameter. We did not find changes in the total Src expression in mesenteric arteries of different calibers but the density of high-affinity ouabain binding sites significantly increased with increasing arterial diameters. We suggested that the increase in mesenteric artery diameter is associated with increased expression of ouabain-sensitive Na,K-ATPase in the vascular wall. This leads to larger Src kinase phosphorylation upon ouabain binding in arteries with larger diameter leading to sensitization of the contractile machinery to [Ca^2+^]_i_.

### 3.1. Ouabain-Induced Potentiation of Contraction Increases with Arterial Diameter

Previous studies, where an acute potentiating effect of ouabain on agonist-induced contraction was tested, demonstrated a broad variation of this potentiating effect [22,24,40]. Importantly, ouabain potentiated primarily arterial sensitivity to contractile agonist [5,22,26,27,42] although this effect was not always seen [43]. The mesenteric vascular bed is an arcading system that contains many branch orders and therefore arteries with different diameters, which are often generalized as mesenteric small arteries [44]. These different-order-branches vary in their dimensions, structure, and function [43], and this variability can affect experimental results.

Different significance of the Na,K-ATPase for structure and function of third and fourth order braches of rat mesenteric artery (~320 µm vs. ~165 µm inner diameter at 70 mmHg) has previously been suggested [4,43]. In contrast to the present study, Zhang and coauthors [43] suggested an increased importance of the Na,K-ATPase in fourth order vs. third order arteries based on the changes of myogenic response in ouabain-induced hypertension [43]. Accordingly, we have previously reported that downregulation of the Na,K-ATPase α2 isoform in rat mesenteric arteries (inner diameter over 200 µm) has different consequences for agonist-induced contraction and pressure-induced myogenic tone, namely, suppression and potentiation, respectively [24]. Moreover, Zhang and coauthors [43] reported that low concentrations of ouabain have no significant effect on agonist-induced contraction in fourth order mesenteric arteries, which is consistent with this study. In accordance with other reports [5,22,26,27,40,42,45], we found that ouabain potentiated the sensitivity to noradrenaline in arteries with inner diameter over 200 µm and, for the first time, suggested the mechanistic background for this.

### 3.2. Sensitization to [Ca^2+^]_i_ Increases with Arterial Diameter

The potentiation of agonist-induced contraction by ouabain was previously ascribed to inhibition of the electrogenic activity of the Na,K-ATPase [1,15] that leads to membrane depolarization and increase in Ca^2+^ influx. It has also been suggested that an accumulation of intracellular Na^+^ upon inhibition of the Na,K-ATPase affects the activity of the Na^+^,Ca^2+^-exchanger and this reduces Ca^2+^ efflux, increases Ca^2+^ load and agonist-induced contraction [1,46]. However, in the present study, no significant effect of 10 µM ouabain on resting [Ca^2+^]_i_ was found. Consistently, ouabain in concentration up to 10 µM did not produce any steady-state [Ca^2+^]_i_ [22,24,25,30] and [Na^+^]_i_ [16,23] increase in previous reports. Only transient potentiation in [Ca^2+^]_i_ in response to agonist stimulation was reported previously in vascular smooth muscles [16,47,48] possibly due to activation of sarcoplasmic reticulum Ca^2+^ transport. These findings suggest an involvement other than modulation of Ca^2+^ influx/release pathways in ouabain-dependent potentiation of vascular sensitivity to contractile agonists.

### 3.3. Ouabain-Induced Src Phosphorylation Increases with Arterial Diameter and Is Associated with an Increase in High-Affinity Ouabain Binding Sites

We have recently shown that 10 µM ouabain increase Src kinase phosphorylation in the vascular wall [22,25,31]. This is consistent with reports from other tissues [32,33,34,35,36,37,38], where the Na,K-ATPase was shown to be integrated in several signaling pathways, including Src kinase signaling [49]. Auto-phosphorylation at Y418 activates Src kinase, which can initiate many signaling pathways. We have previously shown that in arteries this leads to MYPT1 phosphorylation and sensitization to [Ca^2+^]_i_ [22,25].

The conventional pathway for sensitization of smooth muscle cells to [Ca^2+^]_i_ is via Rho kinase translocation and phosphorylation of MYPT1 [50]. We have recently shown that this pathway is significantly potentiated by ouabain [22,25]. Although phosphorylation of MYPT1 was not measured in the present study, we found ouabain-dependent Src phosphorylation associated with increased noradrenaline sensitivity, which strongly supports the previously suggested signaling [22,25]. In the previous study, the effects of noradrenaline and ouabain on Src phosphorylation were additive [22]. Consistent with this we also found in the present study that noradrenaline alone activated Src kinase. However, this activation did not depend on diameter, which was not surprising since the total Src level was similar in arteries of different caliber.

In the present study, we for the first time showed that ouabain-induced sensitization to [Ca^2+^]_i_ in smooth muscles correlates with arterial diameters from 150 µm to 350 µm and this was associated with a corresponding change in phosphorylation of Src kinase. We found that this was not because total Src expression was dependent on artery diameter, but could be explained by an increased density of high-affinity binding sites for ouabain. We suggest that the increase in density of ouabain-sensitive Na,K-ATPase in smooth muscle cell membranes leads to stronger activation of Src signaling upon ouabain stimulation and, thus, sensitization to [Ca^2+^]_i_ and contraction.

We used in the present study 10 µM ouabain that in rodent arterial wall should inhibit only the ouabain-sensitive Na,K-ATPase α2 isoform. This is consistent with the apparent importance of α2 isoform for ouabain-dependent potentiation of arterial contraction [5,16,20,24,51]. Some previous studies in other organs and cell types do not support a role of the α2 isoform in Src signaling. These studies show a distinct role of the α1 isoform [36,52]. However, both α1 and α2 isoforms of the Na,K-ATPase have been shown to interact with Src kinase in skeletal muscles [53]. Moreover, a previous study also suggested activation of Src kinase through the Na,K-ATPase α2 isoform in vascular smooth muscle cells [29]. Thus, it remains to be studied whether the α2 isoform controls Src signaling in the vascular wall or it is mediated through the α1 isoform. One of the possibilities could be that inhibition of the α2 isoform changes the local Na^+^ concentration, which, in turn, shift the Na,K-ATPase α1 isoform into its E2 conformation [54] and, thus, releases the Src kinase [33].

## 4. Significance

The present study suggests that circulating endogenous ouabain [9] can differently modulate resistance arteries of different diameter. Our results suggest that the major effect of ouabain will be seen in mesenteric branches with larger diameter. Smaller arterioles are sparsely innervated [55] and their resistance is mainly controlled by metabolic factors and vaso-reactive substances in the blood [56]. Principle arteries of inner diameter above 200 µm are densely innervated and provide significant changes in resistance upon activation of the sympathetic nervous system [57]. The present study suggests that sensitivity to noradrenaline is predominantly regulated by ouabain in these principle arteries.

This might be especially important for hypertension where ouabain has been suggested to play a role [2]. The mechanism behind its action remains unclear but ouabain elevates vascular tone and peripheral resistance [58]. Taking into account the present findings, we suggest that ouabain increases peripheral resistance primarily via the potentiation of the sensitivity of principle arteries to noradrenaline released from perivascular sympathetic nerves. Although, the importance of ouabain-induced potentiation of myogenic tone in smaller arterioles should not be underestimated [43].

## 5. Methods

All experiments conformed to guidelines from the European Convention for the Protection of Vertebrate Animals used for Experimental and other Scientific Purposes and were approved by and conducted with permission from the Animal Experiments Inspectorate of the Danish Ministry of Environment and Food (# 2016-15-0201-00982 from 01.07.2016).

### 5.1. Isometric Force and [Ca^2+^]_i_ Measurements

Male Wistar Hannover rats, 9–11 weeks of age were obtained from Janvier laboratories (France) and were sacrificed with CO_2_ inhalation after a week of acclimatization. The mesentery was dissected out into ice-cold physiological salt solution (PSS) and branching arteries of different orders were dissected out under microscope. Arterial segments of ~2 mm were mounted in an wire myograph for recording of isometric force (Danish Myo Technology A/S, Aarhus, Denmark) as described previously [24,25,30]. The arterial segment was equilibrated for half an hour at 37 °C in PSS before the passive tension-length curve was constructed [59]. The internal circumference at 100 mmHg was estimated based on this passive tension-length curve (IC100). The IC100 values were used to estimate diameters the arteries would have had in vivo under a transmural pressure of 100 mmHg. These diameters were used in this study to compare the caliber of different arteries.

The arteries were than stretched to a values corresponding 0.9 times of IC100 to perform isometric force measurements. At these settings these arteries are known to develop near-maximal active force upon agonist stimulation [59]. Force (in mN) was recorded with a PowerLab 4/25–Chart7 acquisition system (ADInstruments Ltd., Dunedin, New Zealand) and converted to wall tension by dividing the force with twice segment length (in N/m). The experimental protocol was initiated with the standard-start procedure, where arteries were stimulated 3 times with 10 µM noradrenaline and then one times with 10 µM noradrenaline in high potassium PSS (K-PSS).

For simultaneous measurements of isometric force and [Ca^2+^]_i_, arterial segments mounted and normalized in a myograph were loaded with 2.5 µM fura 2-acetoxymethyl ester (fura 2-AM) dissolved in DMSO with 0.1% (*wt*/*vol*) cremophor and 0.02% (*wt*/*vol*) pluronic F127 for 2 h. Ratiometric [Ca^2+^]_i_ measurements were obtained as described previously [22]. Shortly, arteries were excited alternately at 340 and 380 nm by a 75 W xenon light source, and emitted light was measured at 515 nm. Background fluorescence (after the quenching with 20 mM of MnCl_2_) was subtracted from the measurements. Fluorescence was collected (Felix32 software, ver. 1.2, Photon Technology, Edison, NJ, USA) and the relative changes in [Ca^2+^]_i_ were expressed as the ratio of fluorescence during excitation at 380 nm and 340 nm.

### 5.2. Semi-Quantification of Total Src and Phosphorylated Src in Mesenteric Artery Wall

Segments of rat mesenteric arteries were dissected and mounted in a myograph as described above. The passive tension-length curve measurements were made and IC100 was detected. After the standard-start procedure, arteries were fixed in ice-cold 4% paraformaldehyde (PFA) for 20 min under resting conditions or after 5 min stimulation with either noradrenaline (1 µM) or after 15 min pre-incubation with ouabain (10 µM) followed by 5 min stimulation with noradrenaline (1 µM) in the presence of ouabain. After fixation, arteries were stored in phosphate-buffered saline (PBS) at 4 °C for the following staining.

On the same day, the arteries were placed in PBS with 0.3% Triton-X100 and 1% of bovine serum albumin (BSA) for incubation at 4 °C with antibodies either against total Src or against phosphorylated Src. After 48 h incubation, arteries were washed (3 × 10 min) in PBS with Triton-X100. Arteries stained for phosphorylated Src were then directly used for measurements. To detect phosphorylated pY418 Src, we used primary rabbit monoclonal antibody labelled with Alexa Fluor 647 (Abcam [Catalog nr. ab201860], diluted 1:200). To detect total Src, we used primary Santa Cruz Biotechnology Inc. (Dallas, TX, USA) mouse monoclonal antibody [Catalog no. sc-8056], diluted 1:200. Arteries stained for total Src were, after washing out the primary antibody, incubated for 1 h at room temperature in the dark with the secondary antibody (donkey anti-mouse antibody labeled with Alexa Fluor 555, ThermoFisher Scientific [Catalog no. A-31570], diluted 1:1000). Immediately before measurements, arterial segments were exposed for 10 min to SYTO-16 (diluted 1:1000, Invitrogen, Carlsbad, CA, USA [Catalog nr. S7578]) to stain for nuclei.

After washing, arterial segments were transferred to the confocal microscope (LSM-5 Pascal Exciter, Zeiss, Jena, Germany) and excited alternately at 488 nm (for detection of SYTO-16) and at 543 nm (for detection of total Src) or 560 nm (for detection of phosphorylated Src). The emission signals at 505–530 nm (SYTO-16), 560 nm (total Src), and over 650 nm (phosphor-Src) were stored on the computer and analyzed for fluorescence intensity with ImageJ (National Institutes of Health, Bethesda, MD, USA). Z-stack images with 1 µm interval were collected through the vascular wall. Fluorescence intensities from smooth muscle cells in the arterial wall media were measured and normalized to intensities from arteries stained without primary antibody. The optical slice was fully contained within the media of all vessels as evidenced from SYTO-16 staining, where only smooth muscle cell nuclei, and not endothelial and fibroblasts nuclei, were seen in one section.

### 5.3. Semi-Quantification of Ouabain-Binding Sites in the Arterial Wall

Rat mesenteric arteries were dissected out, mounted in a myograph and IC100 determined as described above. Arteries were then stained with 10 µM BODIPY FL Ouabain (Invitrogen, Carlsbad, CA, USA [Catalog nr. B23461]) for 15 min and SYTO-82 (diluted 1:1000, Invitrogen, Carlsbad, CA, USA [Catalog nr. S11363]) for 10 min to stain for nuclei. Using the confocal microscope a Z-stack of BODIPY FL Ouabain fluorescence intensity was obtained (excitation at 488 nm and emission at 505–530 nm). The localization to medial smooth muscle cells was verified with SYTO-16 staining (as described above). Images were analyzed with ImageJ (National Institutes of Health, Bethesda, MD, USA). Background fluorescence from the arterial wall without BODIPY FL Ouabain was subtracted. For negative control, arteries were first exposed to 1 mM ouabain for 30 min and then BODIPY FL Ouabain was added for another 15 min. This reduced collected fluorescence intensity by approximately 67%.

### 5.4. Solutions and Chemicals

PSS composition was as follows (in mM): 119 NaCl, 3.0 KCl 1.18 KH_2_PO_4_, 1.17 MgCl_2_, 25.0 NaHCO_3_, 0.026 EDTA, and 5.5 glucose, gassed with 5% CO_2_ in air and adjusted to pH 7.4. K-PSS was prepared as PSS, where NaCl was substituted with equimolar KCl. PBS contained (in mM) 137 NaCl, 2.7 KCl, 8.2 Na_2_HPO_4_, 1.8 KH_2_PO_4_, at pH 7.4. If not otherwise indicated, all chemicals were purchased from Sigma Aldrich (Brøndby, Denmark). Ouabain stock solutions were prepared on the day of experiment (minimum 2 h prior to application) in a concentration of 0.01 M in water.

## 6. Data Analysis

GraphPad Prism software (v.7.03 for Windows, GraphPad Software, La Jolla, CA, USA) was used for graphing and statistical analysis. All data are presented as mean values ± SEM. Concentration–response curves were fitted to experimental data using four-parameter, non-linear regression curve fitting. From these curves, logEC_50_ (where EC_50_ is the concentration required to produce a half-maximal response) and maximal response were derived and compared using an extra sum-of-squares *F* test. The correlations between arterial size and other measured parameters, or between changes in [Ca^2+^]_i_ and wall tension were analysed by using goodness-of-fit of linear regression (R^2^, where R is the Pearson correlation coefficient). Linear regression curves were compared by their slopes using one-way ANOVA followed by Bonferroni post-test. A probability (*p*) level of <0.05 was considered significant and *n* refers to number of rats.
